# Characterization of molecular scores and gene expression signatures in primary breast cancer, local recurrences and brain metastases

**DOI:** 10.1186/s12885-019-5752-8

**Published:** 2019-06-07

**Authors:** Mariana Bustamante Eduardo, Vlad Popovici, Sara Imboden, Stefan Aebi, Nadja Ballabio, Hans Jörg Altermatt, Andreas Günthert, Rolf Jaggi

**Affiliations:** 1 Department for Biomedical Research, University of Bern, Murtenstrasse, 40, 3008 Bern, Switzerland; 2 RECETOX, Faculty of Science, Masaryk University, Brno, Czech Republic; 3 Department of Gynecology and Obstetrics, Inselspital, Bern University Hospital, University of Bern, Bern, Switzerland; 4 Department of Medical Oncology, Cantonal Hospital of Lucerne, Lucerne, Switzerland; 5 Breast Center Unit at gyn-zentrum, Lucerne, Switzerland; 6 consultarif DRG, Lohn-Ammannsegg, Switzerland

**Keywords:** Breast cancer, Molecular risk scores, Local recurrence, Brain metastasis, PAM50 subtypes, Gene expression measurement, Immunohistochemistry, RNA isolation and processing, Hierarchical clustering

## Abstract

**Background:**

Breast cancer is a leading cause of cancer-related death in women worldwide. Despite extensive studies in all areas of basic, clinical and applied research, accurate prognosis remains elusive, thus leading to overtreatment of many patients. Diagnosis could be improved by introducing multigene molecular scores in standard clinical practice. Several tests that work with formalin-fixed tissue have become routine. Molecular scores usually include several genes representing processes, response to oestrogens, progestogens and human epidermal growth factor receptor 2 (Her2), respectively, which are combined additively in single values. These multi-gene scores have the advantage of being more robust and reproducible than single-gene scores. Their utility may be further enhanced by combining them with classical diagnostic parameters. Here, we present an exploratory study comparing the RISK and research versions of Oncotype DX recurrence score (RS), Prosigna Risk of Recurrence (ROR) and EndoPredict (EP) with respect to their prognostic potential for ipsilateral recurrence and/or distant relapse in brain, and we compared the scores to the intrinsic subtypes based on PAM50.

**Methods:**

RNA was extracted from formalin-fixed, paraffin-embedded (FFPE) tissue cores of primary tumours, local recurrences and brain metastases. Gene expression was measured on a NanoString nCounter Analysis System. Intrinsic subtypes and molecular scores were computed according to published literature and RISK, RS, ROR and EP were compared against each other and to the intrinsic subtypes Luminal A (lumA), Luminal B (lumB), Her2-enriched (Her2↑), Basal-like (basal), and Normal-like (normal) of PAM50. Local recurrences and brain metastases were compared to their corresponding primary tumours.

**Results:**

All four molecular scores were highly correlated. Highest correlations were observed among genes related to proliferation while lower correlations were found among oestrogen-related genes. The scores were significantly higher in primary tumours progressing to brain metastases as compared to recurrence-free primary tumours and primary tumours that relapsed as local recurrences.

**Conclusions:**

RISK and ROR-P are prognostic for primary tumours metastasizing to the brain. All four scores, RISK, RS, EP and ROR-P failed to discriminate between primary tumours that remained recurrence-free and primary tumours relapsing as local recurrences.

**Electronic supplementary material:**

The online version of this article (10.1186/s12885-019-5752-8) contains supplementary material, which is available to authorized users.

## Background

In spite of early detection, accurate classification and optimal treatment, about 30% of patients with early breast cancer will suffer a locoregional or a distant recurrence [1]. Recurrent breast cancer can develop in essentially any organ of the body; metastases are more aggressive than the primary tumour and account for the majority of deaths related to breast cancer. Many locoregional recurrences can be controlled initially, but they are associated with a high risk of distant metastasis and death [2, 3]. Traditionally, several parameters are related to the risk of recurrence and to the response to drug therapy, e.g. tumour size, histological grade and proliferation, lymph node involvement, lymphovascular invasion, overexpression and/or amplification of the human epidermal growth factor receptor 2 (Her2) gene and failure to express the oestrogen receptor (ER) and progesterone receptor (PR). More recently, several robust molecular scores have been developed. Each score is based on a rather simple algorithm integrating expression levels of several genes. Such scores are correlated with prognosis and their values are more stable than expression values of individual genes.

Starting with the seminal work of Perou et al. [4] and Sørlie et al. [5], through iterative refinements, a set of stable molecular subgroups (intrinsic subtypes) of breast cancer was identified from microarray data with thousands of genes. Fifty genes were sufficient to reliably classify breast cancer into the five intrinsic subtypes: Luminal A (lumA), Luminal B (lumB), Her2-enriched (Her2↑), Basal-like (basal) and Normal-like (normal) [6, 7]. The intrinsic subtypes are important parameters as they added independent prognostics (survival of patients) [4, 5] to the classical risk factors and predictors [6, 7]. The PAM50 was further developed to a commercial test made by NanoString under the brand Prosigna. It identifies the intrinsic subtype and provides the risk of (distant) recurrence (ROR-P) [8]. The Oncotype DX® recurrence score (RS) provides information about the risk of distant recurrence [9, 10] as well as the benefit of chemotherapy in ER+/Her2- breast cancer patients [11]. The RS assay has been clinically validated and the test is performed at the central clinical reference laboratory of Genomic Heath, Inc. EndoPredict (EP) and EPclin, which is a combination of EP with tumour size and nodal status, predict distant recurrence and they can be applied to ER+/Her2- breast cancers [12]. EP also predicts response to chemotherapy in ER+/Her2- breast cancer patients [13] and is available as a commercial test as well. The RISK score predicts disease free survival in patients with ER+ breast cancer [14]. Even though these scores combine genes related to proliferation and ER, the overlap between them is reduced or non-existent. The RISK and RS also contain two genes overexpressed in Her2↑ tumours: *ERBB2* (the gene coding for Her2) and *GRB7* (the gene is linked to *ERBB2* on chromosome 17). While multigene molecular risk scores have improved the prediction of distant recurrence, their ability to predict ipsilateral local recurrence has remained uncertain.

In this study, we compared four multigene risk scores (research versions of RISK, RS, ROR-P and EP) and contrasted them with the intrinsic subtypes built from PAM50. The scores of recurrence-free patients and patients who later developed an ipsilateral local recurrence or a brain metastasis were compared, as well as the expression pattern between primary tumours and their matched local recurrences or brain metastases. Our results revealed that RISK, RS, ROR-P and EP show an amazingly similar performance with respect to their risk of recurrence. All four scores failed completely to discriminate between primary breast cancer that remained recurrence-free (curated control patients) and primary tumours that relapsed and formed locally recurrent tumours.

## Methods

### Patient population

Archival material was collected retrospectively from formalin-fixed paraffin-embedded (FFPE) tumour samples from the repository of the Swiss Sentinel Node Study and the Biobank Bern. Eighty-seven samples were from primary tumours, 43 from patients without local or distant recurrence (controls); 25 from patients who developed local recurrence in the residual breast parenchyma in the same quadrant and 19 from patients who developed brain metastasis. In addition, tumour tissues derived from 20 local recurrences and 25 brain metastases were obtained from the Biobank Bern. Primary tumours and local recurrences were available for 19 patients, while primary tumours and brain metastases were available also for 19 patients. Median follow-up time of recurrence-free patients was 88 months. The study was approved by the Cantonal Ethics Committee of the Canton Bern (Ref 2017–02025). Primary tumours were diagnosed between 1987 and 2009. Tumours were removed by breast conservative surgery and treated postoperatively with radiotherapy. Patients with uncertain or positive resection margins were excluded from the study. Patients were treated with chemotherapy and adjuvant treatment depending on hormone receptor status and tumour stage.

### Immunohistochemistry (IHC)

The ER and the PR were measured immunohistochemically using 1% labelling as threshold for negative (−) and positive (+) staining. The Her2 was measured immunohistochemically. Staining was defined as negative (Her2 = 0 or 1+) or positive (Her2 = 3+); tumours with partial membrane staining of more than 10% tumour cells (Her2 = 2+) were reanalysed by Fluorescence In Situ Hybridization (FISH), according to the ASCO-CAP guidelines [15]. Proliferation was measured with a monoclonal antibody against Ki-67 (MIB-1) and expressed as labelling index (LI). As thresholds for low and high Ki-67, LI < 14% and LI ≥ 14% were used, according to Cheang and coworkers [16].

The IHC-based classification was used to build immunohistochemical surrogates of intrinsic subtypes as described by Guiu et al. [17]. Each tumour was assigned to one of the four classes: lumA (ER+ and/or PR+, Her2– and Ki-67 LI low), lumB (ER+ and/or PR+, Her2– and Ki-67 LI high, or ER+ and/or PR+, Her2+ and Ki-67 LI low or high), Her2↑ (ER–, PR– and Her2+) and basal (ER–, PR– and Her2–). Normal-like tumours were not considered for this classification. The PAM50-based intrinsic subtype prediction was performed as described [18]. The RISK and the risk of recurrence, ROR-P, were determined from all the 132 tumours, the Oncotype DX recurrence score, RS, and EndoPredict, EP, were determined from ER+ and Her2– tumours (based on IHC).

### RNA isolation and processing

Two core punches of FFPE material were prepared based on a local inspection of tumour blocks, the paraffin was dissolved, and the RNA subjected to demodification and purification on silica-based columns (AmpTec GmbH, Hamburg, Germany), as described previously [19, 20]. Seven samples were discarded due to poor quality of RNA or insufficient recovery. The remaining 132 RNAs were used for gene expression analysis.

Eighty-four test and twelve reference genes were measured on a NanoString nCounter System (NanoString Technologies, Seattle USA). The genes are listed in additional file 1. Eight negative controls (no homology to eukaryotic RNA) and six positive probes (directed against unrelated RNA) were also included. The RNAs corresponding to the positive probes (spike-in RNAs) are present in each sample before hybridization. One hundred ng RNA was hybridized overnight at 65 °C and processed on an nCounter Prep Station. Gene expression was then quantified with a Digital Analyzer. The procedure is completely enzyme-free and highly robust regarding RNA fragmentation. The raw signal for each gene was background corrected and normalized using the negative and positive control genes in each sample. The resulting data was highly reproducible and NanoString data correlated with microarray data or real-time PCR data [21]. The expression data was further normalized against internal reference genes and log2 transformed to determine the intrinsic subtypes and the molecular scores of each sample (see below). Raw data is shown in additional file 3.

### Normalization of gene expression data

The raw expression data from the Digital Analyzer was normalized using nSolver version 3.0 (NanoString Technologies, Seattle USA) or NanoStringNorm R package [22]. Both analyses gave the same results (data not shown). Briefly, the expression data was background corrected and poor-quality samples were excluded. The data of all the remaining 132 samples were normalized using all the 12 reference genes (reference genes are listed in additional file 1). The normalized data were then subjected to a hierarchical cluster analysis [23] based on the union of 74 genes of PAM50, RISK, RS and EP in nSolver (version 3.0) using Euclidean distance.

The PAM50 and the molecular scores were originally developed from microarray data or from real-time PCR data. Erroneously, the probes for *CDC6* and *CDCA1* which are part of PAM50 classifier were missing in the CodeSet during hybridization. To assess the effect of the two genes on the stability of PAM50 predictions, we compared the subtype assignments made by PAM50 classifier (R package genefu with robust standardization [18]) on two publicly available data sets [24] [25] with and without the two genes. Globally, < 3% of samples (7 from [24] and 3 from [25], respectively) changed their subtype when *CDC6* and *CDCA1* were discarded. Thus, we considered that the assignment without the two genes was stable enough to assign all 87 primary and 45 recurrent cancers to one of the five classes: normal, lumA, lumB, Her2↑ and basal [18].

### Analysis of molecular scores

The NanoString data was normalized separately for each molecular score using the procedures and reference genes described for each score: three reference genes for RISK [14], five reference genes for RS [10] and three reference genes for EP [12]. The genes for ROR-P are part of PAM50; therefore, normalized PAM50 data was used. Threshold cutoffs and re-scaling of RS (0–100) were omitted as the scores were exclusively used for relative comparison with other scores or with PAM50 subtypes. The RISK, RS and EP were computed from at least two groups of functionally related genes, where the first group comprised genes reflecting the proliferative potential of tumour cells (termed PRO-subscore), and the second group integrated the capacity of tumour cells to respond to steroid hormones and their receptors (oestrogen and progesterone and their receptors). They were termed ER- and PR-subscore. A PRO-subscore was also computed from the ROR-P score [6], based on the proliferation-related genes in PAM50.

### Statistical analysis

The intrinsic subtypes were determined based on immunohistochemical data [17] and on gene expression [18]. The agreement between the IHC and PAM50 classifications was measured in terms of Cohen’s kappa statistic. Only the tumours with lumA, lumB, Her2↑ or basal classification were included for this comparison, since normal-like tumours were not mapped to an IHC-defined subtype.

The agreement between scores was estimated with Cohen’s kappa statistics categorizing one-third of samples into high risk and two-thirds of samples into intermediate-low risk. The significance level was set a priori to *p* = 0.05 for all tests. The correlations between molecular scores and subscores were evaluated using Spearman’s correlation test.

Each molecular score was compared within PAM50 intrinsic subtypes (lumA, lumB, Her2↑, basal and normal) using the Mann-Whitney test. Similarly, molecular scores and subscores were compared between groups of samples (primary tumours from controls, primary tumours from patients who developed local recurrence, primary tumours from patients who developed brain metastasis, local recurrences and brain metastases).

## Results

### Comparison of gene expression and immunohistochemistry

This study is based on 87 primary breast cancers, 20 local recurrences and 25 brain metastases. Forty-three primary tumours remained recurrence free, 19 patients had a local recurrence and 25 had a brain metastasis. Clinical characteristics of patients and histological data of primary and recurrent tumours are summarized in Table 1 together with immunohistochemical data on the ER, the PR, Her2 and Ki-67 (Table 1).

**Table 1:** Patient and tumour characteristics

Group	Control	Local recurrence		Brain metastasis	
		primary local	recurrence	primary distant	metastasis
Nr. of patients (pairs)	43	26 (19 pairs)		25 (19 pairs)	
Total number of tumours	43	25	20	19	25
Median age (range)	55 (30–90)	60 (28–85)		56 (35–74)	
Menopausal category					
Pre-menopausal	15	7		9	
Post-menopausal	27	18		10	
Unknown/missing	1	0		0	
Tumour grade					
Grade 1	4	1	1	0	
Grade 2	29	18	10	8	
Grade 3	10	6	3	11	
Unknown/missing	0	0	6	0	
IHC parameters					
ER status					
Positive	37 (86%)	20 (80%)	17 (85%)	6 (32%)	7 (28%)
Negative	6 (14%)	5(20%)	3 (15%)	13 (68%)	18 72%)
PR status					
Positive	34 (79%)	16 (64%)	12 (60%)	5 (26%)	5 (20%)
Negative	9 (21%)	9 (36%)	8 (40%)	14 (74%)	20 (80%)
Her2 status					
Positive	4 (9%)	4 (16%)	2 (10%)	11 (58%)	14 (56%)
Negative	37 (86%)	20 (80%)	17(85%)	7 (37%)	11 (44%)
Unknown/missing	2 (5%)	1 (4%)	1 (5%)	1 (5%)	0
MIB-1					
< 14%	19 (44%)	10 (40%)	6 (30%)	4 (21%)	0
≥ 14%	24 (56%)	14 (56%)	13 (65%)	14 (74%)	25 (100%)
Unknown/missing	0	1 (4%)	1 (5%)	1 (5%)	0
IHC subtype					
lumA	18 (42%)	9 (36%)	6 (30%)	0	0
lumB	17 (40%)	10 (40%)	10 (50%)	6 (32%)	7 (28%)
Her2↑	1 (2%)	3 (12%)	1 (5%)	8 (42%)	11 (44%)
basal	5 (12%)	2 (8%)	2 (10%)	4 (21%)	7 (28%)
Unknown/missing	2 (5%)	1 (4%)	1 (5%)	1 (5%)	0
PAM50 subtype					
normal	2 (5%)	3 (12%)	0	1 (5%)	0
lumA	17 (40%)	9 (36%)	6 (30%)	3 (16%)	0
lumB	14 (33%)	8 (32%)	9 (45%)	2 (11%)	4 (16%)
Her2↑	3 (7%)	4 (16%)	3 (15%)	9 (47%)	15 (60%)
basal	7 (16%)	1 (4%)	2 (10%)	4 (21%)	6 (24%)

Main characteristics of patients and tumours in the following groups: Controls (recurrence-free tumours), Local recurrence (primary tumours and local recurrences) and Brain metastasis (primary tumours and brain metastases). The size of primary and recurrent tumours was not available.

In a first comparison, the RNA-based expression of ER (*ESR1*), PR (*PGR*) and Her2 (*ERBB2*) was compared to the immunohistochemical data for the respective proteins (additional file 2, panels A-C). Similarly, the expression of Ki-67 (measured with the probe *MKI67*) was compared to the proliferation marker Ki-67. The Ki-67 was quantified as labelling index (LI) with a monoclonal antibody against Ki-67 (MIB-1) (additional file 2, panel D). The Ki-67 was dichotomized into low (LI < 14% immunostaining with MIB-1) and high (LI ≥ 14% immunostaining with MIB-1) as described previously [16]. The comparison of immunohistochemistry data and gene expression data revealed excellent concordance between all four markers.

Intrinsic subtypes were also built from IHC data of the tumours [17] (Table 1). The procedure is described in the Methods section. The subtypes according to IHC were compared to subtypes according to gene expression (PAM50). The agreement between the two classifications was only moderate (Cohen’s kappa, κ = 0.58). The discordances between classifications were 33, 49, 17 and 18% for lumA, lumB, Her2↑ and basal subtypes, respectively. Tumours with normal-like classification were omitted from this comparison.

### Hierarchical clustering of primary breast cancer, local recurrences and distant metastases

**Fig. 1:**
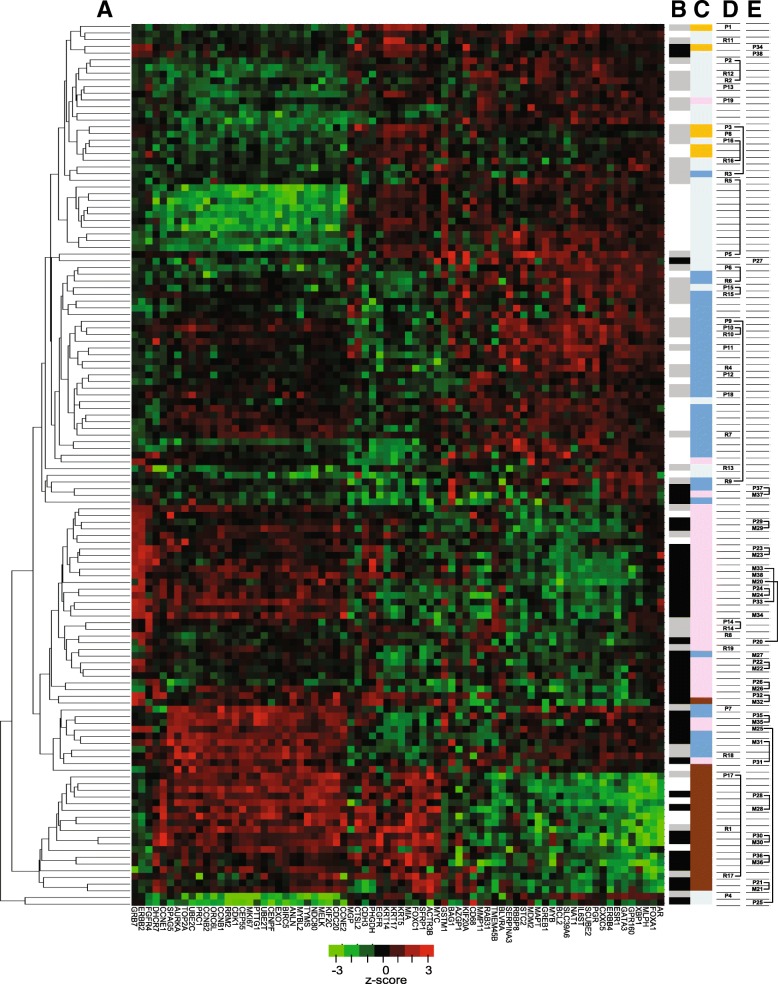
Hierarchical clustering of primary tumours, local recurrences and distant metastases. **a** The gene expression profile of all tumours was characterized by hierarchical clustering and the results are shown as a heatmap. Rows correspond to the 132 tumour samples and columns correspond to the 74 genes (which are part of RISK, RS, EP and/or PAM50). **b** The panel indicates for each sample whether it is a control (white), a primary tumour that later relapsed as local recurrence (grey) or a primary tumour that later progressed to a brain metastasis (black). The same colours are used for the local recurrences and brain metastases (grey and black, respectively). **c** Indicates the intrinsic subtype of each tumor: normal-like (ochre), lumA (light blue), lumB (blue), Her2↑ (pink) or basal (brown). **d** Indicates 19 pairs of primary tumours (P1 – P19) and local recurrences (R1 – R19). Pairs clustering in the vicinity of each other are highlighted with brackets. **e**. Indicates 19 pairs of primary tumours and brain metastases. Primary tumours and corresponding metastases are labeled P20 – P38 and M20 – M38, respectively. Pairs of tumors clustering in the vicinity of each other on the heatmap are depicted with brackets

Primary tumours, local recurrences and distant metastases were then characterized on the basis of gene expression. Gene expression was measured with NanoString and the raw data was normalized using 12 reference genes, representing a combination of five reference genes for PAM50, three for RISK, five for RS and three for EP. The test and reference genes are listed in additional file 1. The 132 tumours were characterized by hierarchical clustering using all the 74 genes represented in PAM50, RISK, RS and/or EP. The result is shown as a heatmap (Fig. 1, panel a), with genes by columns and tumours by rows. Tumours that remained recurrence-free were termed controls, and are marked with white boxes in panel B; primary tumours that relapsed as local recurrence and local recurrences are marked with grey boxes (panel B); and primary tumours that progressed to brain metastases and brain metastases are marked with black boxes (panel B). Recurrence-free controls and local recurrences (primary tumours and recurrences) predominantly clustered in the upper part of the heatmap while primary tumours metastasizing to the brain and brain metastases are more abundant in the lower part (panel B). Intrinsic subtypes [7] are symbolized with colours (panel C) according to Parker et al. [7]: normal (ochre), lumA (light blue) and lumB (blue), Her2↑ (pink) and basal (brown). Normal and lumA are predominantly in the upper part, Her2↑ and basal in the lower part of the heatmap. The majority of lumB are between these two clusters.

Twenty-five tumours were primary tumours that progressed to local recurrences and 20 were local recurrences. Corresponding tissue from primary and recurrence tumours was available from 19 patients marked as P1 – P19 for primaries and R1 – R19 for recurrences (panel D). The analysis of these pairs of tumours showed that, to the extent of genes considered here, they were not particularly similar, with only three pairs clustering together.

Similarly, among the 44 primary tumours and brain metastases 19 were matched samples, primary tumours are labelled P20 – P38 and metastases were labelled M20 – M38 (panel E). It may be worth mentioning that 11 pairs of primary metastases from the same patients clustered in the immediate vicinity of each other on the heatmap (panel E). This clustering may suggest that, at least with regard to the expression of the genes considered here, primary tumours and corresponding brain metastases are more similar to each other than primary tumours and local recurrences. It is also apparent that primary tumours with enriched Her2 tend to metastasize to the brain.

### Comparison of molecular scores

**Fig. 2:**
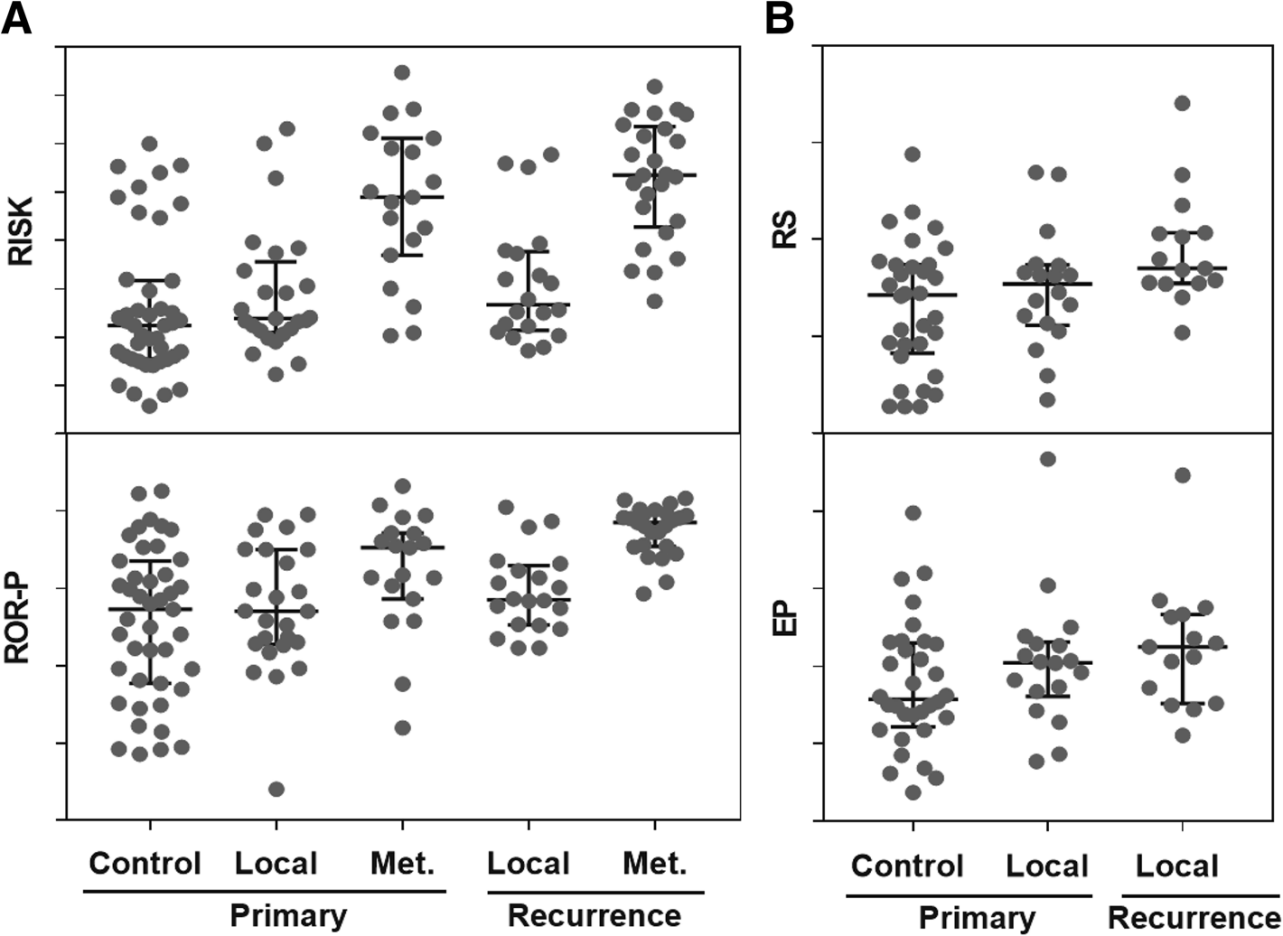
RISK, ROR-P, RS, and EP scores in primary tumours and recurrences. **a** Scatter dot plots (median with interquartile range) of RISK and ROR-P scores are shown for primary tumours (Controls, Local, Met.) and for recurrences (Local and Met.) The total number of tumours was 132. **b** Scatter dot plots (median with interquartile range) of RS and EP scores are shown for 72 ER+/Her2- primary tumours (Control and Local) and recurrences (Local)

The gene expression data was also used to compute the four molecular scores RISK, RS, ROR-P and EP according to the published instructions, using the test and reference genes described for each of them [6, 10, 12, 14] (see Additional file 1 for the complete list of genes). The distributions of scores are shown separately for control tumours and primary tumours that later relapsed as local recurrence or as brain metastasis. Similarly, the distributions of scores are shown for local recurrences and brain metastases. The data for RISK and ROR-P are based on all available tumours (Fig. 2a). The calculation of RS and EP is limited to ER+ and Her2- tumours (Fig. 2b). Only three primary tumours later relapsed as brain metastases and four brain metastases were ER+ and Her2-, therefore, the corresponding data for these tumours are not shown. For all four molecular scores, no statistically significant differences were found between recurrence-free controls and primary tumours that later relapsed as local recurrences (Mann-Whitney test) (Table 2). Recurrence-free controls were also compared to primary tumours that later relapsed as brain metastases. The RISK and ROR-P scores were higher in tumours that relapsed as compared to recurrence-free controls (*p* <  0.01) and primary tumours that relapsed as local recurrence (p <  0.01 for RISK and *p* = 0.03 for ROR-P). The discrimination between control tumours and primary tumours of brain metastases was also evident at the level of the three subscores representing the hormonal status (ER-, PR and Her2-subscores). The molecular scores were also compared between primary tumours and local recurrences and clearly, they did not change over time (Table 2). Similarly, the RISK score did not increase between primary tumours and brain metastases, whereas the ROR-P score increased during progression from primary tumours to brain metastases (*p* = 0.01).

**Table 2:** Comparison of molecular scores between control tumours, local recurrence and brain metastasis

		Local recurrence		Brain metastasis	
		primary local	recurrence	primary distant	metastasis
RISK	**control**	0.16	0.03	< 0.01	< 0.01
	**primary local**		0.52	< 0.01	< 0.01
	**recurrence**			< 0.01	< 0.01
	**primary distant**				0.34
RS	**control**	0.45	0.01	–	–
	**primary local**		0.09	–	–
	**recurrence**			–	–
	**primary distant**				–
ROR-P	**control**	0.54	0.16	< 0.01	< 0.01
	**primary local**		0.35	0.03	< 0.01
	**recurrence**			0.35	< 0.01
	**primary distant**				0.01
EP	**control**	0.2	0.05	–	–
	**primary local**		0.36	–	–
	**recurrence**			–	–
	**primary distant**				–

RISK and ROR-P scores were calculated from all 132 samples, RS and EP scores from 72 ER+/Her2- samples as described. Scores in each group were compared using the Mann-Whitney test. Shown are *p*-values of all pairwise comparisons. Two-tailed *p* values > 0.05 were considered not significant.

**Fig. 3:**
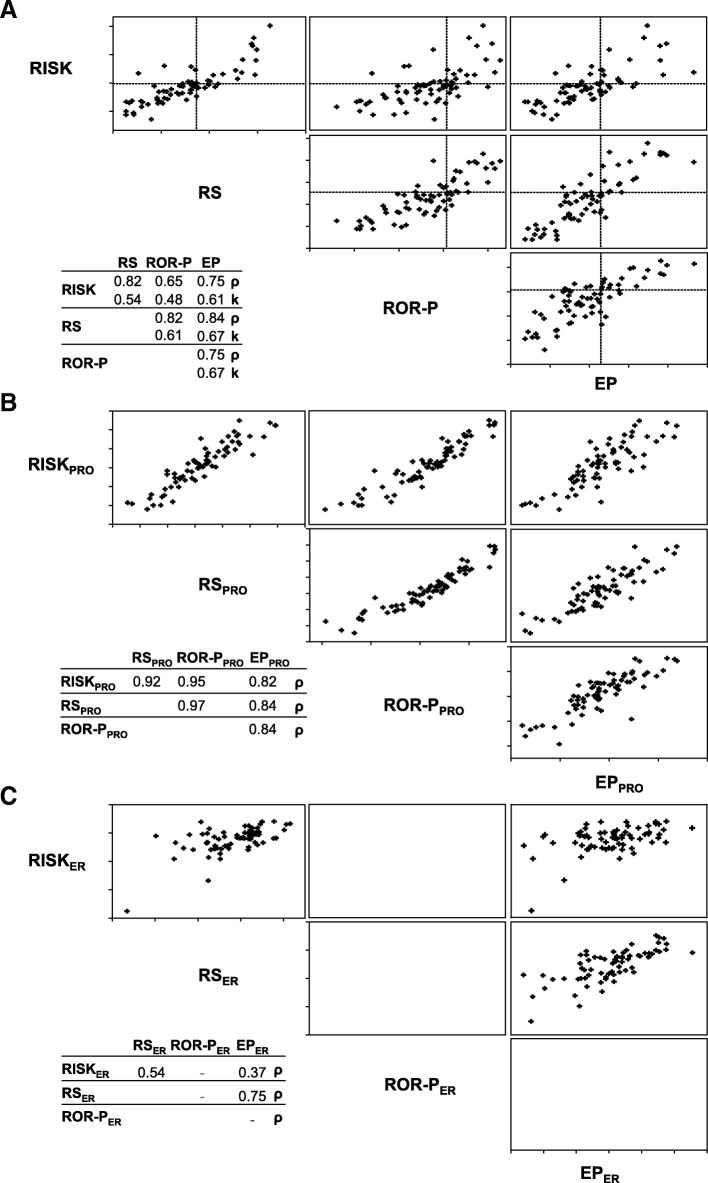
Comparison of molecular scores. **a** Molecular scores were determined from primary tumours and pairwise comparisons are shown as scatter plots. Spearman’s correlation coefficients (ρ) and Cohen’s kappa (κ) are shown in the inset. For κ, low and intermediate scores (2/3 of samples) were compared to high scores (1/3 of samples). **b** Genes related to proliferation were used to determine proliferation-related subscores of RISK (RISK_PRO_), RS (RS_PRO_), ROR-P (ROR-P_PRO_) and EP (EP_PRO_). Pairwise correlations of subscores are plotted and Spearman’s correlation coefficients (ρ) of subscores are shown in the inset. **c** Similarly, ER-subscores were calculated from ER-related genes in RISK (RISK_ER_), RS (RS_ER_) and EP (EP_ER_). Pairwise comparisons of subscores and Spearman’s correlation coefficients (ρ) are shown

The four scores were directly compared against each other using correlation plots (Fig. 3a). Depending on the different comparisons the Spearman’s correlations were 0.65–0.84 and the Cohen’s kappa statistics (for dichotomized versions – see Methods) indicated moderate to substantial agreement (κ within 0.48–0.67). The results indicate remarkable similarities between any two scores and at the same time it is obvious that the correlations are remarkably high, although in fact they are never very high.

The correlation of all the pairs of scores was divided into PRO- and ER-subscores and analysed separately (see Methods for further details). Increased expression of genes related to proliferation produced higher PRO-subscores indicating poorer prognosis of the patient. Higher ER-subscore correlates with a favourable response to steroid hormone-based therapies and therefore correlates with better prognosis. The PRO-and ER-subscores were determined for RISK, RS and EP (Fig. 3b and c). Spearman correlations for each of the comparisons were very strong (ρ ≥ 0.82), while correlations between ER-subscores were considerably lower (ρ = 0.37–0.75).

### Relationship between intrinsic subtypes and molecular scores in primary and recurrent breast cancer

**Fig. 4:**
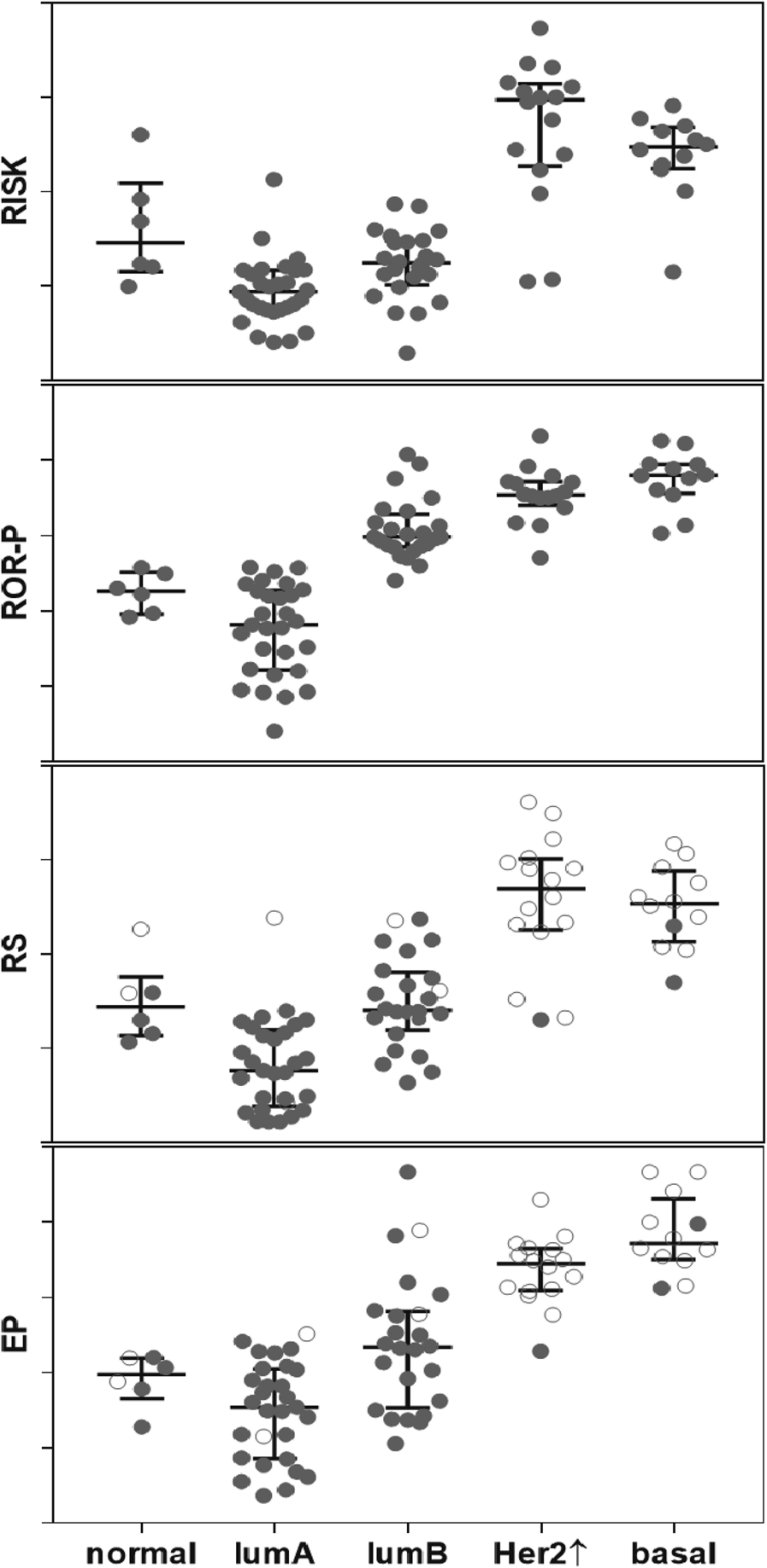
Allocation of molecular scores among PAM50 intrinsic subtypes. Molecular scores were measured from 87 primary cancers (including control tumours and primary tumours from patients who developed local recurrences or brain metastases). Scatter dot plots (median with interquartile range) of RISK, ROR-P, RS, and EP are shown itemised into intrinsic subtypes normal, lumA, lumB, Her2↑ and basal. The RS and EP were only validated for ER+/Her2- tumours, therefore, ER- and/or Her2+ tumours are shown exclusively for comparison (open circles)

As implied by the IHC-based definition of intrinsic subtypes, the PAM50 subtypes were aligned with the molecular scores, in the sense that the scores generally increased from lumA to lumB, Her2↑ and basal, respectively. In Fig. 4, the classification into normal, lumA, lumB, Her2↑ and basal subtypes is shown separately for RISK, ROR-P, RS and EP. Since the RS and EP were developed for ER+ and Her2– tumours, and the tests were not validated for ER– and/or Her2+ tumours, the respective scores are marked distinctly. The scores were lowest for lumA tumours and significantly higher in lumB tumours. The scores of lumA and lumB tumours were significantly lower than the scores of Her2↑ and basal subtypes (Table 3) which were similar. The molecular scores of normal tumours tended to be higher for lumA tumours. The comparison between normal and lumB led to a less clear picture: the molecular scores were higher in lumB tumours than in normal-like tumours, with respect to ROR-P, they were not different with respect to the three other scores (Table 3).

**Table 3:** Allocation of molecular scores among PAM50 intrinsic subtypes

		lumA	lumB	Her2↑	basal
RISK	**normal**	< 0.01	0.17	0.01	0.02
	**lumA**		< 0.01	< 0.01	< 0.01
	**lumB**			< 0.01	< 0.01
	**Her2↑**				0.1
ROR-P	**normal**	0.05	< 0.01	< 0.01	< 0.01
	**lumA**		< 0.01	< 0.01	< 0.01
	**lumB**			< 0.01	< 0.01
	**Her2↑**				0.07
RS	**normal**	0.04	0.43	–	–
	**lumA**		< 0.01	–	–
	**lumB**			–	–
	**Her2↑**				–
EP	**Normal**	0.32	0.25	–	–
	**lumA**		< 0.01	–	–
	**lumB**			–	–
	**Her2↑**				–

RISK, and ROR-P were calculated from all 87 primary tumours, RS and EP from all 31 ER+/Her2- primary tumours as described. Scores were compared between any two groups using the Mann-Whitney test. Shown are *p*-values of all pairwise comparisons. Two-tailed *p* values > 0.05 were considered not significant.

## Discussion

Multigene molecular risk scores aim to stratify patients into risk of recurrence groups. In this study we measured the expression of genes of the PAM50 signature and four prognostic scores (RISK, RS, ROR-P and EP) in primary breast cancer, in local recurrences and in brain metastases*.* We compared primary tumours from patients that remained recurrence-free (controls) with primary tumours that relapsed as local recurrences or primary tumours that relapsed as brain metastases, and we compared primary tumours with matched local recurrences or brain metastases.

We are aware of the limitations of our study, since the patients in our cohort were selected retrospectively and based on availability of tumour material. Thus, the hypotheses generated in this study should be verified with material from prospectively collected patient data or, preferably, in a controlled clinical trial. Invariably, the recurrence-free patients had lower risk scores than those who developed distant metastases.

### Comparison of molecular and immunohistochemical classification

Primary tumours were classified separately into intrinsic subtypes on the basis of molecular and IHC data. The comparison revealed that the agreement between molecular and IHC classification was only ‘moderate’ (κ = 0.58; Table 1). In a similar study, Chia and colleagues compared 347 tumours [26]. Although they observed a slightly higher concordance between molecular and histological classifications (κ = 0.64), the two classifications were characterized by numerous misclassifications [26]. The results of both studies suggest that molecular and immunohistochemical classifications are related but clearly not interchangeable.

### Hierarchical clustering of primary tumours, local recurrences and distant metastases

Primary tumours, local recurrences and brain metastases were further characterized by hierarchical clustering (Fig. 1), revealing that most tumours with normal-like and lumA subtypes were separated from Her2↑ and basal subtypes, while lumB tumours were situated between the two clusters. A similar arrangement was described in the two original studies that were based on 65 breast cancers of 42 individuals [4, 5] or 78 tumours and cDNA microarrays with more than 8.000 genes [4, 5].

We also compared gene expression profiles of primary tumours with profiles of corresponding recurrences from the same patients. Local recurrences were also preferentially of luminal subtype, like the corresponding primary tumours, but the majority of matched samples were noticeably unrelated and did not separate in the immediate vicinity on the heatmap (Fig. 1). Our results probably do not allow drawing further conclusions, but it seems remarkable that many changes take place at the level of gene expression between primary tumours and local recurrences that arose later in the same area of the breast. Other studies based on IHC concurrently reported that more than 70% of primary tumours and local recurrences were lumA or lumB [27, 28].

In contrast, primary tumours that relapsed as brain metastases were predominantly Her2↑ or basal (nine and four of 19, respectively) and matched samples often had the same intrinsic subtype. Moreover, 11 metastases cluster next to their matched primary tumours on the heatmap. Similar results were reported from a study based on 20 pairs of primary tumours and brain metastases. Seventeen metastases retained the same PAM50 subtype as their corresponding primary tumours and 12 primary tumours and metastases clustered close to each other [29]. The comparison was based on 143 genes, 61 of which were the same as in our study. Other results based on IHC showed that primary tumours with Her2-enriched or basal subtype preferentially relapsed as brain metastases [30, 31].

### Comparison of molecular scores

Primary tumours that relapsed as brain metastases had significantly higher scores than primary tumours that remained recurrence-free (controls) (Fig. 2 and Table 2). The results are shown for RISK and ROR-P. The RS and EP were not included, as these scores were developed and validated in ER+ tumours with normal Her2. In addition, ER-, PR- and Her2- subscores from RISK were also able to discriminate between tumors that relapsed as brain metastases and recurrence-free controls. The four molecular scores were also compared between recurrence-free controls and primary tumours that relapsed locally. We observed that molecular scores did not increase when primary tumours relapsed as local recurrence (Fig. 2, Table 2). This contradicts some earlier reports that the molecular scores were higher in primary tumours that relapsed as local recurrences when compared to controls [32–34]. However, the different definitions of local recurrence (e.g. recurrences in the chest skin after mastectomy or any site in the breast were considered local recurrence) may explain this apparent discrepancy. It cannot be excluded that such tumours have higher scores than local recurrences according to our definition. In addition, none of these earlier observations was validated in independent retrospective or prospective studies. Moreover, several groups failed to find a molecular signature in primary tumours discriminating between controls and tumours relapsing as local recurrence. For instance, Servant and colleagues tested 22 different gene-signatures, including RS and found that none of them was able to predict ipsilateral local recurrences [35]. In this study we tested RS and other three additional scores and, similarly, found no difference between recurrence-free controls and primary tumours that later reappeared as local relapse. Therefore, patients at risk of local recurrence are probably missed by the current molecular scores. In fact, a de novo search for such markers might be successful as we observed two genes, *RBBP8* and *MLL3*, from our list of 84 genes whose expression was different in primary tumours of local recurrences as compared to controls.

The RISK, RS, ROR-P and EP scores were originally developed as prognostic parameters [6, 10, 12, 14]. Although each score is computed from different genes, their prognostic power seems to be similar and, it is possible that they can be used interchangeably. They are all built from genes associated with proliferation; the RISK, RS and EP scores contain additional genes related to ER and the response to oestrogen. The *ERBB2* gene which codes for Her2, and the *GRB7* gene are part of RISK and RS. These two genes are physically linked on chromosome 17 and overexpression correlates with amplification of DNA in this region of the chromosome.

The correlations between any two molecular scores were 0.65 < ρ < 0.85 (Fig. 3a). While all the correlations between PRO-subscores (genes related to proliferation) were higher (0.82 ≤ ρ ≤ 0.97; panel B), the correlations between ER-subscores (genes related to ER response) were clearly lower (0.37 ≤ ρ ≤ 0.75; panel C). The implications of the lower correlations among ER-subscores are not obvious and it should be clarified whether ER-subscores could be improved by adding or replacing some genes that are not consistently related to ER and its response.

Our analyses are based on retrospectively collected tumour samples. The results must thus be considered cautiously, but they seem to indicate that the proliferation-related genes (PRO-subscore) favour the agreement between RISK, RS, ROR-P and EP, while the ER-subscores are more variable and therefore limit the agreement between the scores.

Previous studies compared RS and EP [36], or RS and ROR [37]. In these comparisons, RS was measured in the laboratories of Genomic Heath. As a consequence, the analyses and comparisons were based on separate tissue sections, and RNAs were processed with different assays and on different instruments. Nevertheless, the studies reported similar results. We directly compared RISK with RS that was either measured at Genomic Health, or in our own laboratory, and we found higher correlations when RS was measured on the same tissue and in the same laboratory (ρ = 0.82, κ = 0.52 as compared to ρ = 0.43, κ = 0.23). The comparison was based on 220 prospectively collected samples (data not shown and [38]).

The study presented here comprised 87 primary tumours (43 recurrence-free controls, 25 primary tumours of local recurrences and 19 primary tumours of brain metastases). Each tumour was classified into intrinsic subtypes according to PAM50. Twenty-nine tumours were lumA; these tumours had the lowest scores, corresponding to the best prognosis (Fig. 4). Twenty-four tumours were lumB; the corresponding scores were significantly higher than in lumA (*p* < 0.01 for all scores). Sixteen primary tumours were Her2↑ and 12 were basal, the corresponding RISK and ROR-P scores were higher than lumB tumours (p < 0.01) (Fig. 4, Table 3). This observation is consistent with other studies [8] showing the lowest scores for lumA tumours and higher scores in all other subtypes.

## Conclusions

In this study we measured four molecular scores – RISK, RS, ROR-P and EP – in recurrence-free primary breast cancer (controls) and in primary tumours that later relapsed with a local recurrence or a brain metastasis. All four scores performed similarly, and high pairwise correlations were found between them. None of the four scores allowed discriminating between recurrence-free controls and primary tumours that relapsed with a local recurrence. This observation is in conflict with other studies that reported that RS and EP are prognostic for both, local and distant recurrences. Our data confirmed that primary tumours that later relapsed with a brain metastasis had significantly higher RISK and ROR-P scores. As many of these tumours were Her2↑, they did not qualify for testing with RS and EP. We found that primary tumours and corresponding brain metastases were similar (at least with respect to gene expression). This is in contrast to local recurrences where gene expression profiles of primary tumours and corresponding local recurrences were surprisingly different. The results may imply that tumour cells that survive from primary tumours undergo major changes when they proliferate and form local recurrences. This observation should be considered when molecular scores derived from primary tumours are used to estimate the risk of recurrence, or even guide decisions on potential therapies.

## Additional files

Additional file 1: Gene Identifications, Categories and Score affiliations. The list contains all the genes (gene symbols and accession numbers). The column marked “Score” indicates for each gene whether it is part of PAM50 (1), RISK (2), RS (3) and/or EP (4). ROR-P is part of PAM50. Reference genes are marked (Ref). (XLS 32 kb)

Additional file 2: Comparison of IHC and gene expression in primary tumours. **A**, Primary tumours were dichotomized into ER-negative (ER–) and ER-positive tumours (ER+) based on antibody staining using 1% labelling as threshold. The relative gene expression of *ESR1* (mRNA for ER) was plotted for each tumour (y-axis). Similarly, the gene expression of *PGR* was plotted for primary tumours dichotomized into PR-negative (−) and PR-positive (+) tumours using 1% labelling as threshold. **C**, The Her2-negative (Her2–) and Her2-positive tumours (Her2+) were compared to *ERBB2* expression. **D**, Finally, the proliferation marker Ki-67 was quantified with MIB1 antibody and cells were dichotomized based on the labelling index (LI). The LI high was defined high when LI ≥ 14% and low for LI < 14%. The LI was plotted against the mRNA coding for Ki-67, MKI67 (relative expression levels of *ESR1*, *PGR*, *ERBB2* and *MKI67* after log2 transformation). Boxplots show interquartile ranges, whiskers go to 2.5 and 97.5 percentiles, dots are used for outliers. Statistical evaluations were performed using the Mann-Whitney test. The *p*-values (two-tailed) for each analysis are shown for each plot. (PDF 34 kb)

Additional file 3: Raw data from NanoString experiment. This spreadsheet contains the raw nCounter data used for the comparisons. (XLS 4183 kb)

## Data Availability

The raw data from NanoString is available in Additional file 3.

## Abbreviations

Her2↑: Her2-enriched
basal: Basal-like
EP: EndoPredict
ER: Oestrogen receptor
FFPE: formalin-fixed paraffin-embedded
FISH: Fluorescence In Situ Hybridization
Her2: Human epidermal growth factor receptor 2
IHC: Immunohistochemistry
LI: labelling index
lumA: Luminal A
lumB: Luminal B
normal: Normal-like
PR: Progesterone receptor
ROR: Risk of recurrence
RS: Recurrence score
