# Supplementary figures and images for: Characterization of molecular scores and gene expression signatures in primary breast cancer, local recurrences and brain metastases
Source: BMC Cancer. 2019 Jun 7;19:549. doi: 10.1186/s12885-019-5752-8 (PMC6556009; doi:10.1186/s12885-019-5752-8)

Additional file 2

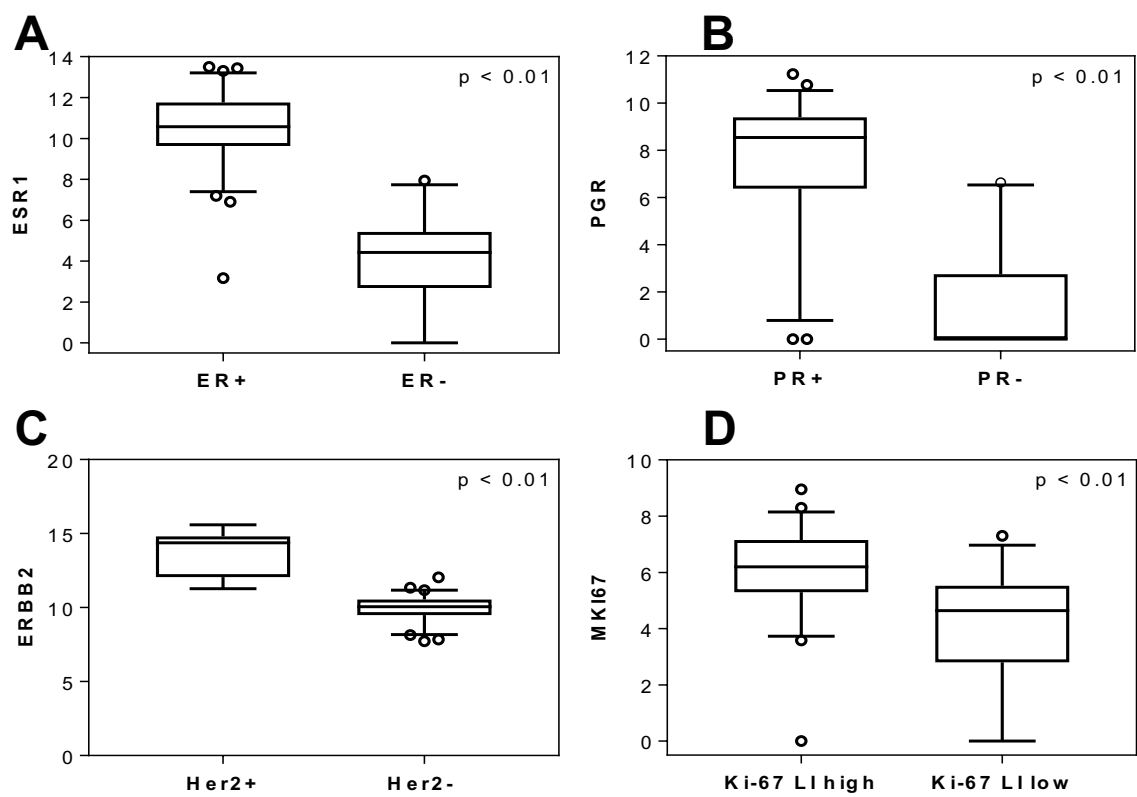

Supplement: Supplementary file 2 — Comparison of IHC and gene expression in primary tumours. A, Primary tumours were dichotomized into ER-negative (ER–) and ER-positive tumours (ER+) based on antibody staining using 1% labelling as threshold. The relative gene expression of ESR1 (mRNA for ER) was plotted for each tumour (y-axis). Similarly, the gene expression of PGR was plotted for primary tumours dichotomized into PR-negative (−) and PR-positive (+) tumours using 1% labelling as threshold. C, The Her2-negative (Her2–) and Her2-positive tumours (Her2+) were compared to ERBB2 expression. D, Finally, the proliferation marker Ki-67 was quantified with MIB1 antibody and cells were dichotomized based on the labelling index (LI). The LI high was defined high when LI ≥ 14% and low for LI < 14%. The LI was plotted against the mRNA coding for Ki-67, MKI67 (relative expression levels of ESR1, PGR, ERBB2 and MKI67 after log2 transformation). Boxplots show interquartile ranges, whiskers go to 2.5 and 97.5 percentiles, dots are used for outliers. Statistical evaluations were performed using the Mann-Whitney test. The p-values (two-tailed) for each analysis are shown for each plot. (PDF 34 kb) [file 12885_2019_5752_MOESM2_ESM.pdf]
